# Energy Management Strategy of a Hybrid Power System Based on V2X Vehicle Speed Prediction

**DOI:** 10.3390/s21165370

**Published:** 2021-08-09

**Authors:** Ming Ye, Jing Chen, Xu Li, Kai Ma, Yonggang Liu

**Affiliations:** 1 Key Laboratory of Advanced Manufacturing Technology for Automobile Parts, Ministry of Education, Chongqing University of Technology, Chongqing 400054, China; cqyeming@cqut.edu.cn (M.Y.); ChenJing@2019.cqut.edu.cn (J.C.); 929212925@2019.cqut.edu.cn (K.M.); 2 State Key Laboratory of Mechanical Transmissions, College of Mechanical and Vehicle Engineering, Chongqing University, Chongqing 400044, China; andylyg@umich.edu

**Keywords:** plug-in hybrid electric vehicle, smart grid connection, deep learning, energy economy, energy management

## Abstract

Energy consumption in vehicle driving is greatly influenced by traffic scenarios, and the intelligent traffic system (ITS) has a key role in solving the real-time optimal control of hybrid vehicles. To this end, a new energy management control strategy based on vehicle-to-everything (V2X) communication for vehicle speed prediction was proposed to dynamically adjust the engine and motor power output according to the traffic conditions. This study is based on intelligent network connectivity technology to obtain forward traffic state data and use a deep learning algorithm to model vehicle speed prediction using the traffic state data. The energy economy function was modeled using the MATLAB/Sinumlink platform and validated with a plug-in hybrid vehicle model simulation. The results indicate that the proposed strategy improves the vehicle energy economy by 13.02% and reduces CO_2_ emissions by 16.04% under real vehicle driving conditions, compared with the conventional logic threshold-based control strategy.

## 1. Introduction

According to the National Telematics Industry Standard System Construction Guidelines published by the Chinese Ministry of Transportation, with the development of intelligent transportation systems, intelligent transportation systems should form a standard system that can support telematics applications and meet the needs of transportation management and services by 2025 [1]. The use of intelligent transportation systems to achieve the real-time optimal control of hybrid vehicles has significantly drawn the attention of scholars. The overall development of hybrid vehicles is closely related to the design of energy management strategies. To develop an optimal energy management strategy, the predicted vehicle speed is used to calculate the future vehicle energy demand, which is used to adjust the vehicle’s power distribution in real time to obtain a suitable fuel economy and thus alleviate the congestion and auto emissions in urban areas [2,3]. Owing to the time-varying nature and high uncertainty of the real driving environment, it is necessary to shift from the prediction of future vehicle speed using historical data or standard operating conditions to accurately predicting the vehicle speed using vehicle networking technologies, as well as Vehicle to Vehicle (V2V) and Vehicle to Infrastructure (V2I) communication technologies to achieve multiple information fusion, such as traffic information and surrounding vehicle driving status [4,5].

Based on three typical working conditions, the key feature parameters were extracted and subjected to principal component analysis in the study of Pan et al. [6]. Yang Yalian et al. established a prediction model based on model prediction with multi-order Markov and neural networks to predict vehicles’ speed, based on which they proposed a method for model prediction of energy management strategy [7].For vehicles with fixed routes, such as buses, speed analysis using Global Positioning System (GPS)/Geographic Information System (GIS) information was used by Li et al. [8] and established a green speed guidance model for buses. Yang et al. used GPS data of cabs to estimate the average speed of a road with cab trajectory data and combined it with neural networks to design a speed prediction model [9]. The abovementioned studies have focused on developing energy management strategies based on speed prediction for fixed operating conditions using GPS information and historical databases. However, in the actual driving process, the vehicle operating conditions are a random and uncertain process, and the control effect of hybrid vehicle energy management under a specific cycle of operating conditions has certain limitations. At present, the most effective method is to use intelligent transportation technology for speed prediction, road information, traffic flow conditions, weather conditions, and real-time control of the vehicle power system to achieve efficient and clean use of fuel. To obtain more accurate speed prediction information and solve the problem of real-time energy management and optimization of hybrid vehicles, this paper proposes a speed prediction method based on V2V and V2I communication. This study comprises three parts: first, a traffic model of a traffic light intersection for urban traffic is established to simulate real-time vehicle speed and traffic information when V2V and V2I communications are available; second, deep learning is used to train multi-step advance speed prediction through the information provided by V2V and V2I communications; finally, based on the predicted speed and current State of charge (SOC) values, a control strategy based on a combination of logic threshold and instantaneous optimization is used to dynamically allocate the demand torque to create a hybrid vehicle with optimal energy economy.

## 2. Hybrid Power Drive Train Modeling

### 2.1. Hybrid Vehicle Model

**Figure 1:**
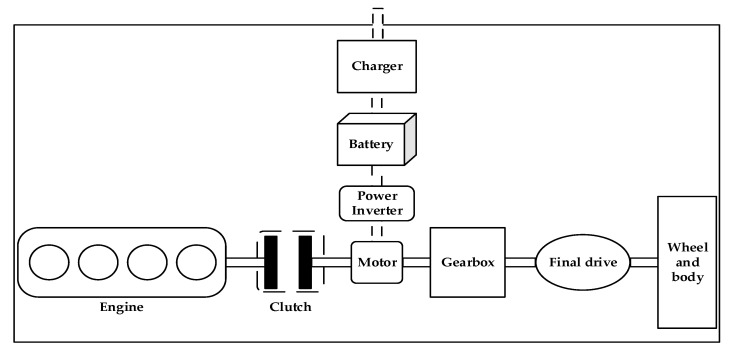
Structure of plug-in single-axis parallel hybrid power system.

In this study, we chose a plug-in single-axis parallel hybrid system as the object of study, and a plug-in single-axis parallel hybrid system structure is shown in Figure 1. From the figure, it can be seen that the engine, clutch, motor, and other key components along the same axis, through the clutch combination, allow only the engine to provide power directly to the vehicle, but also with the motor joint drive; in the clutch disconnected state, the motor alone drives the vehicle or regenerative brakes to charge the battery.

Based on the MATLAB/Simulink simulation platform, a combination of experimental modeling and theoretical modeling was used to model a powertrain model, which consists of the engine, clutch, electric motor, battery and continuously variable transmission (CVT), and main gearbox. According to the main parameters of the vehicle (Table 1), the key components as well as the whole vehicle model are modeled for the research object.

#### 2.1.1. Engine Model

Engine fuel consumption is an important index for evaluating the engine performance and is a key parameter for developing energy management strategies. The engine model in this study was established through bench experiments using the experimental table look-up method.

**Figure 2:**
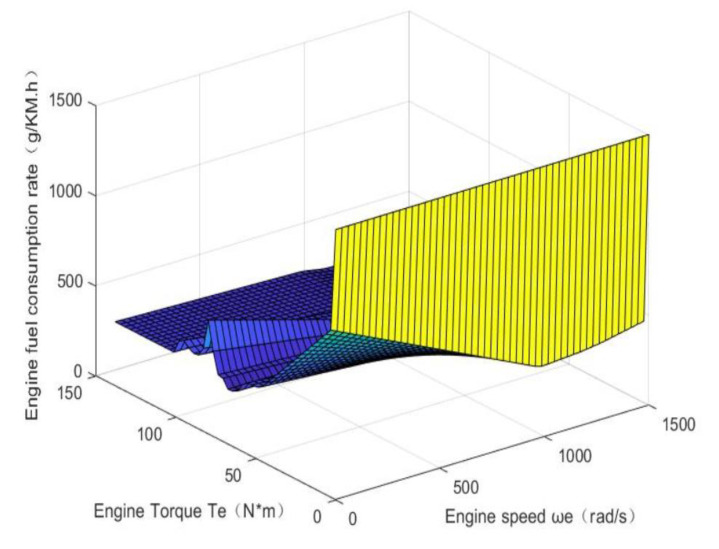
Engine fuel consumption rate.

The fuel consumption of the engine under different loads was measured at different engine speeds [10], and the obtained bench test data were processed using the interpolation fitting method to fit the relationship between the effective fuel consumption rate, speed, and engine torque, as shown in Figure 2.

#### 2.1.2. Motor Model

In this study, a disk-type permanent magnet synchronous motor, which is smaller and lighter than the conventional motor in the hybrid power system, is selected as the second power source. The disk-type permanent magnet synchronous motor is spatially coupled to the magnetic field generated by the three symmetrical currents during torque and power control, in which the three-phase winding armature coordinate system of the stationary stator a, b, c is replaced by the two-phase winding d-q right-angle coordinate system [11]. The dynamic model and motion control equations of the permanent magnet synchronous motor can be introduced after the park transformation, as follows:

(1) $$\begin{array}{l} U_{d}=Ri_{d}+L_{d}\frac{di_{d}}{dt}-p\omega_{m}L_{q}i_{q}\\ U_{q}=Ri_{q}+L_{q}\frac{di_{q}}{dt}-p\omega_{m}L_{d}i_{d}+p\omega_{m}K_{e}\\ T_{m}=1.5pK_{e}i_{q} \end{array}$$

(2) $$\frac{d\omega_{m}}{dt}=\frac{T_{m}-B\omega_{m}-T_{L}}{J}$$

where *R* is the resistance (Ω); $U_{d}$ and $U_{q}$ are the phase voltages (V) of the d- and q-axes, respectively; $i_{d}$ and $i_{q}$ are the phase currents (A) flowing through the d and q axes, respectively; $L_{d}$ and $L_{q}$ are the inductances (H) generated by the d-and q-axes, respectively; $\omega_{m}$ is the rotor angular velocity (rad/s); p is the number of electrode pairs; $K_{e}$ is the counter-electromotive force coefficient (V/rad∙s^−1^); $T_{L}$ is the load torque (Nm); *B* is the viscous frictional assisted damping; and *J* is the motor rotational inertia (kg/m^2^).

**Figure 3:**
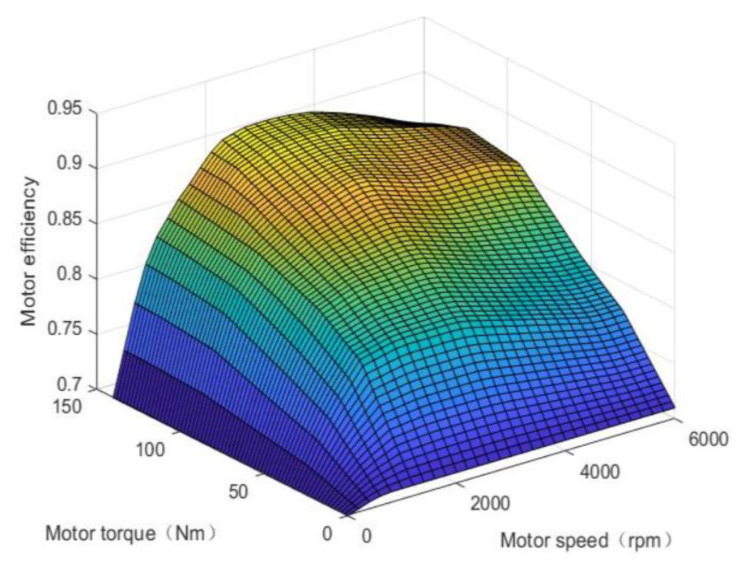
Motor efficiency.

When calculating the power consumption or energy return of the motor, the steady-state interpolation model of the motor is used instead of its cumbersome mechanism formula, thus improving the efficiency of the calculation. The relationship between the speed, torque, and efficiency of the motor is shown in Figure 3.

#### 2.1.3. Battery Model

To simplify the complexity of the battery, in this study, we adopted an internal resistance model, which is the battery equivalent to an open-circuit power supply with an internal resistance in series in the circuit; it is a model based on experimental data that equates the battery to an ideal voltage source in series with a resistance, ignoring the effect of temperature on the battery.

**Figure 4:**
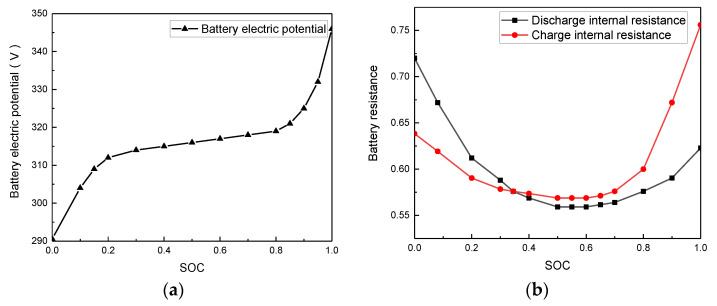
Battery model, (**a**) Battery internal resistance model; (**b**) Battery electric potential model.

From the charge/discharge test on the battery, the relationship curve between the battery electric potential charge/discharge internal resistance and the battery SOC can be derived, as shown in Figure 4a,b.

#### 2.1.4. Transmission Model

The mathematical model of the master and slave pulleys of the transmission is expressed as:

(3) $$\begin{array}{l} T_{\mathrm{cvt}-in}=T_{drep}-I_{z}\omega_{z}\\ T_{cvt-out}=T_{cvt-in}\cdot i_{cvt}\cdot \eta_{cvt}-I_{b}\omega_{b} \end{array}$$

where $T_{cvt-in}$ is the CVT input torque, $T_{cvt-out}$ is the CVT output torque, $T_{drep}$ is the demand torque of the hybrid system, $I_{z}$ is the rotational inertia of the active pulley on the input shaft, $I_{b}$ is the rotational inertia of the driven pulley on the output shaft, $\omega_{z}$ is the active pulley speed, and $\omega_{b}$ is the driven pulley speed.

The CVT speed ratio is calculated by the following formula:

(4) $$i_{cvt}=\frac{\omega_{z}}{\omega_{b}}$$

**Figure 5:**
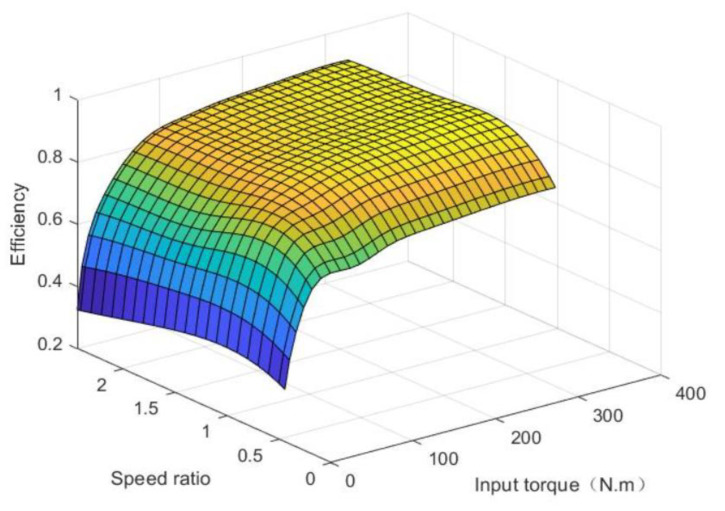
CVT efficiency model.

The CVT efficiency model can be derived by measuring the CVT speed ratio versus torque at the RPM through bench tests. The CVT efficiency model is shown in Figure 5.

#### 2.1.5. Whole Vehicle Model

The driving force analysis of the car shows that the car is only subject to rolling, air, ramp, and acceleration resistances in the driving process. Assuming that the vehicle is driven on a road with slope, α, only the longitudinal dynamics of the vehicle are considered, and the lateral deflection and the handling stability of the vehicle are not considered [12]. The equilibrium equation of the car driving is as follows:

(5) $$F_{w}=mg\cos\alpha +\frac{C_{d}Av_{b}^{2}}{21.25}+mg\sin\alpha +\delta m\frac{dv}{dt}$$

where *A* is the windward area of the vehicle, $C_{d}$ is the air resistance coefficient, *m* is the mass of the car, *g* is the acceleration owing to gravity, *α* is the slope of the road, $v_{b}$ is the speed of the car (km/h), *v* is the speed of the car (m/s), and *δ* is the rotating mass conversion factor.

### 2.2. Traffic Model

**Figure 6:**
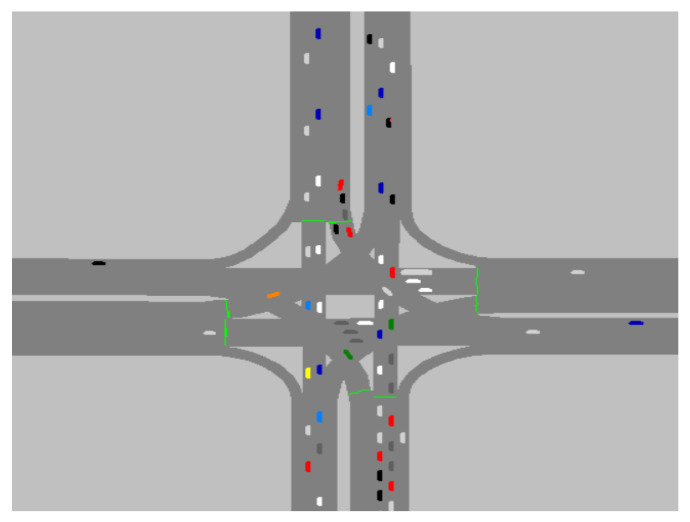
Schematic diagram of traffic scene simulation.

In this study, based on the Wiedemann following model, the traffic simulation software VISSIM was used to establish the urban traffic microscopic traffic model. The constructed simulation scenario is shown in Figure 6, wherein the traffic signal position time and intersection are configured and the traffic flow, vehicle acceleration, and deceleration are set [13,14]. The simulated vehicle obtains the speed and traffic information of the first 30 vehicles in front of the target vehicle through V2X communication technologies to provide data support for speed prediction. Owing to the randomness and diversity of the actual driving cycle, the traffic model can only reflect the route characteristics with strong regularity. In this study, a specific traffic scenario traffic light intersection is selected, and the delay, error rate, road gradient, and collision of vehicle communication are ignored to simplify the traffic model and to facilitate the evaluation of the effectiveness and accuracy of the vehicle speed prediction model [15].

VISSIM can record the driving trajectory, speed, acceleration, and other information of each vehicle in the simulation scenario. The vehicle at 800 m from the traffic light at the intersection is selected as the target vehicle for the study in the simulation scenario, and a data collection point is set every 30 m from the target vehicle to collect the position, speed, and acceleration information of the first 30 vehicles in front of the target vehicle. The obtained data are used as real-time vehicle speed and traffic information obtained through V2V and V2I communication.

## 3. Vehicle Speed Prediction

### 3.1. Vehicle Speed Prediction Model Architecture

**Figure 7:**
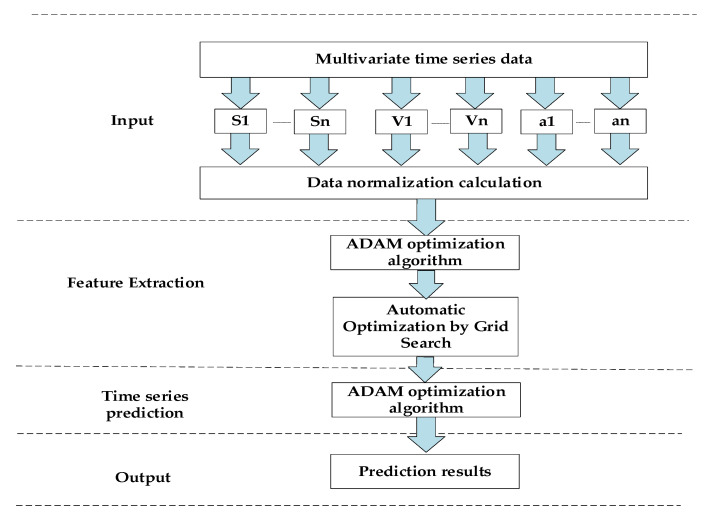
Predictive model framework.

In the process of vehicle driving, due to the change of vehicle speed, range is relatively large, the vehicle driving data passed to the neuron nodes may cause the activation function to exceed the limit, and the network does not converge or converge slowly when the neural network is training, thus causing the accuracy of the network output to decline. Therefore, before the network training, we team data for the normalization process, extracting the mean and standard deviation of multivariate time series arrays of traffic models and using data normalization calculations to speed up the efficiency of training. In this study, two commonly used optimization models, ADAM and SGD, are selected, and then the optimization results are compared; the ADAM optimization model with less errors is selected for training. The model is not only suitable for dealing with sparse gradients but also non-smooth objectives owing to its momentum and adaptive characteristics [16]. The ADAM model was established; the activation function used in this paper is ReLU function, function formula: f(x) = max(0,x). Compared with the sigmod function and the tanh function, it has the following advantages: (1) it overcomes the problem of gradient disappearance and (2) it speeds up the training speed. The biggest problem in deep learning is the gradient disappearance problem, which is especially serious in the case of using saturated activation functions such as tanh and sigmod, while the ReLU function is much faster to train by virtue of its linear, non-saturated form. Using grid search to automatically find hyperparameters, the same set of data is trained by grid search with a different number of hidden layers, number of nodes per layer, learning rate, and number of iterations as variables and root mean square error as an indicator, and the parameter used for the final minimum root mean square error is the optimal parameter for grid search. Each parameter ranges from the number of iterations (10, 100, 1000, 1000), the number of hidden layers [1-5], the number of nodes per layer (8, 16, 32, 64, 128, 256, 512), and the learning rate (0.1, 0.01, 0.001, 0.0001). The prediction model framework diagram is shown in Figure 7.

According to the basic structure and training algorithm of the deep learning network determined in the previous section, the root mean square error of training in ascending order when choosing different numbers of hidden layers and number of neurons is shown in Table 2. The model was run to determine the optimal parameters with two hidden layers, 256 nodes per layer, a learning rate of 0.1, and 100 iterations.

### 3.2. Deep Learning Network

In this study, when a deep learning network is used for training, the input quantity is the position, speed, and acceleration of the first 30 vehicles in front of the target vehicle, and the output quantity is the predicted vehicle speed and acceleration of the target vehicle at different prediction steps [17]. The input and output vectors of the deep learning network can be represented by Equations (6) and (7), respectively.

(6) $$N_{\mathrm{in}}=\left\{S\left(1\right),S\left(2\right),\cdot \cdot \cdot S\left(n\right);V\left(1\right),V\left(2\right),\cdot \cdot \cdot V\left(n\right);a\left(1\right),a\left(2\right),\cdot \cdot \cdot a\left(n\right)\right\}$$

(7) $$N_{out}=\{V(1),V(2),\cdot \cdot \cdot V(p);a(1),a(2),\cdot \cdot \cdot a\left(p\right)\}$$

where $N_{in}$ is the input vector of the deep learning network; $N_{out}$ is the output vector of the deep learning network; *S*(1), *S*(2), ..., *S*(*n*) is the location of the first n vehicles in front of the target vehicle; *V*(1), *V*(2), ..., *V*(*n*) is the speed of the first *n* vehicles in front of the target vehicle; *a*(1), *a*(2), *...*, *a*(*n*) is the acceleration of the first *n* vehicles in front of the target vehicle; *V*(1), *V*(2), *...*, *V*(*p*) is the predicted vehicle speed of the target vehicle when the predicted time domain is *p*; and *a*(1), *a*(*2*), *...*, *a*(*p*) is the predicted acceleration of the target vehicle when the predicted time domain is *p*.

**Figure 8:**
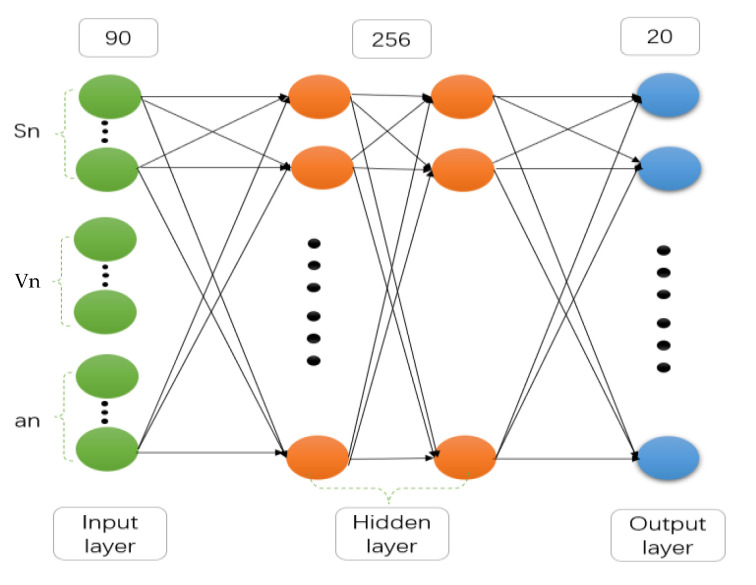
Deep learning network composition.

By training the input of the vehicle driving information in front of the target vehicle and the output of the driving information of the corresponding target vehicle in the future time period, the speed prediction model can be obtained. Provided that the information time series of the first n vehicles in front of the target vehicle is given, the speed information in the prediction time domain of the corresponding target vehicle can be calculated, that is, the speed information of a certain time in the future. The composition of the deep learning network used in this study is shown in Figure 8.

**Figure 9:**
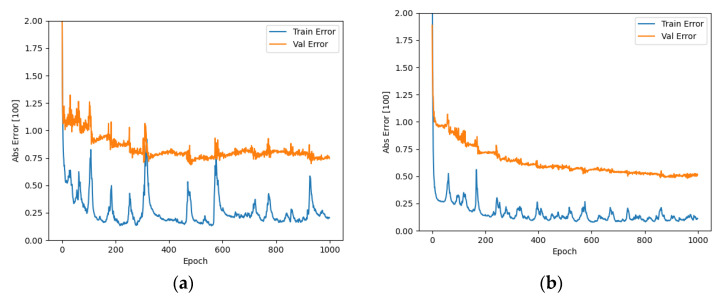
Training set and validation set error (absolute error): (**a**) 3 s training set and validation set error; (**b**) 10 s training set and validation set error.

**Figure 10:**
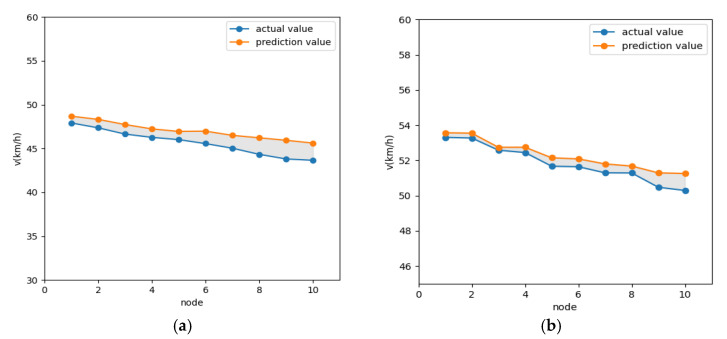
Real speed vs. predicted speed: (**a**) 3 s real speed vs. predicted speed; (**b**) 3 s real speed vs. predicted speed.

**Figure 11:**
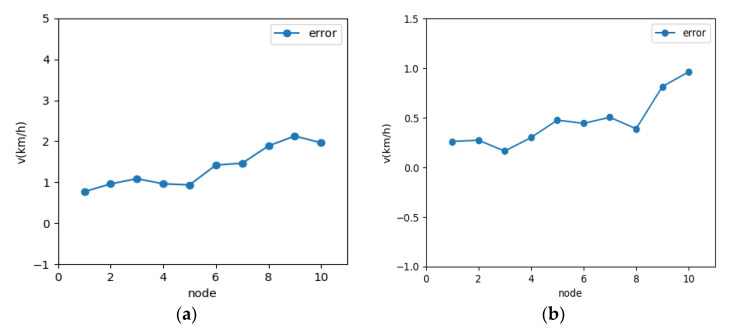
Predicted speed error: (**a**) 3 s prediction speed error; (**b**) 10 s prediction speed error.

The established neural network was trained according to the network structure parameters determined above as well as the training algorithm, and the multiple time series data were divided into training, validation, and test sets in the ratio of 6:2:2. The errors (absolute errors) of the training and test sets in the last five steps are shown in Figure 9. It is shown that the absolute errors are 1.30 and 0.60 smaller for the prediction time domain of 3 s and 10 s, respectively, indicating that the parameters, such as the number of hidden layers and nodes selected by automatic optimization search are reasonable. From Figure 10 and Figure 11, the selected validation dataset, the overall error of the prediction time domain is within [0, 2] for 3 s and within [0, 1] for 10 s. The prediction values obtained using this deep learning network were consistent with the target values, and the performance of the network was suitable. Therefore, the established deep learning network is considered to meet the expected results and can better perform the vehicle speed prediction calculation.

**Figure 12:**
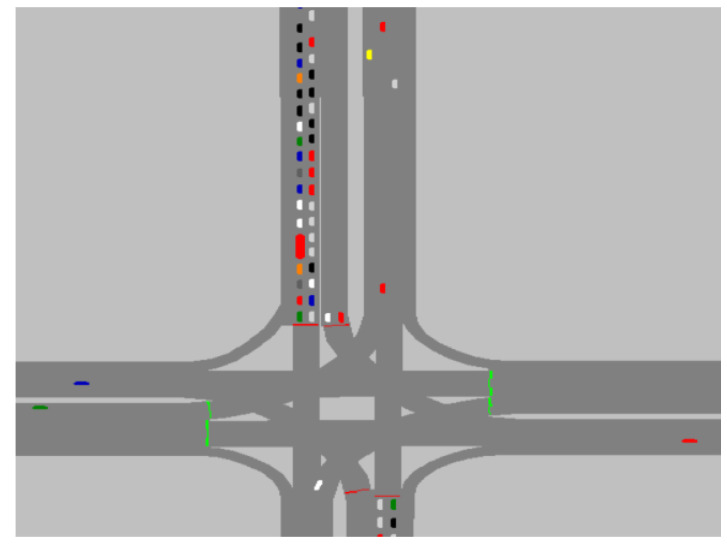
Congestion Scenarios.

**Figure 13:**
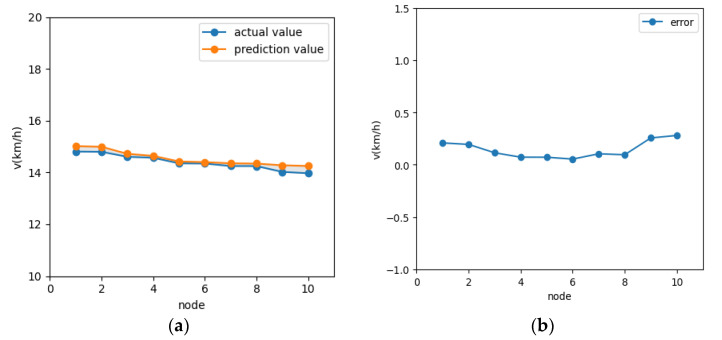
Congestion Scenarios prediction speed: (**a**) actual speed vs. predicted speed; (**b**) prediction speed error.

In order to verify the effectiveness of the speed prediction model, it is shown that the speed prediction model achieves effective prediction, not only in the traffic light scenario, but also in other scenarios, such as congested roads. Traffic flow allocation is set up in VISSIM to build a traffic congestion scenario, as shown in Figure 12. The data collector is set in the road section, and the collected data are input to the vehicle speed prediction model to obtain the prediction results, as shown in Figure 13. Under the congestion scenario, the prediction time domain is 10 s, vehicle speed error is within [0, 0.5] overall, and the prediction accuracy is high.

In two traffic scenarios, the predicted values obtained using this speed prediction model are basically consistent with the actual values; therefore, it is considered that the established speed prediction model, which satisfies the expected results, can perform accurate speed prediction calculations.

## 4. Real-Time Optimized Energy Management Algorithm

### 4.1. Evaluation of Energy Consumption Economy of Plug-In Hybrid Electric Vehicles

The energy management control strategy is aimed at making the energy consumption economically optimal in the predicted time domain to achieve an overall better result. The energy of the plug-in hybrid vehicle comes partly from grid charging and partly from fuel [18]. In this study, the energy consumption economy is used as the objective function to minimize the grid and fuel costs.

(8) $$E_{d}(t)=\int_{0}^{t_{f}}P_{f}Q_{f}(t)+P_{e}\frac{P_{b}(t)}{\eta_{g}}dt$$

Here, $t_{f}$ is the terminal moment; $P_{f}$ denotes the fuel price (L/rmb); $P_{e}$ denotes the price per kWh (KW/rmb); $\eta_{g}$ denotes the grid charging efficiency, which is considered as 0.98; $Q_{f}\left(t\right)$ is the amount of fuel consumed at moment *t*; and $P_{b}\left(t\right)$ refers to the charging and discharging power of the battery at moment *t*, which is greater than 0 when discharging and less than 0 when charging.

(9) $$P_{b}(t)=\frac{T_{m}(t)\omega_{m}(t)}{9550\eta_{m}(t)\eta_{b}(t)}$$

Here $T_{m}\left(t\right)$ and $\omega_{m}\left(t\right)$ are the motor torque and speed at moment *t,* respectively; $\eta_{m}\left(t\right)$ is the motor efficiency at moment *t*; and $\eta_{b}\left(t\right)$ is the battery charging and discharging efficiency at moment t.

In Equation (8), when the powertrain charges the power battery while driving, the energy at this time originates from the fuel rather than the grid; therefore, the energy economy function of this part should be:

(10) $$E_{d}=P_{f}\eta_{t}\frac{P_{b}(t)}{\eta_{g}\eta_{m}(t)}$$

### 4.2. Energy Management Control Strategy

In this study, we apply a control strategy combining logical threshold and transient optimization by obtaining the current vehicle state (such as demand torque and *SOC* value), comparing it with the set logical threshold parameters, evaluating its operation mode, conducting transient optimization in the specified area combined with the vehicle energy economy function, and achieving optimal energy economy in other areas through the threshold value. Finally, the optimal distribution of power source torque in the predicted time domain is achieved.

The logical threshold parameters of the control strategy are vehicle demand torque, maximum engine output torque, optimal engine output torque, and battery *SOC* value, where the change in battery *SOC* value largely causes a change in mode. According to the operating mode and characteristics of the plug-in hybrid vehicle, the constraints that make the target value of the *SOC* meet the normal driving conditions and are as follows:

(11) $$\left\{ \begin{array}{l} SOC_{\min}\leq SOC_{t}\leq SOC_{\max}\\ T_{m-\min}(\omega_{m,t})\leq T_{m,t}\leq T_{m-\max}(\omega_{m,t})\\ T_{e-\min}(\omega_{e,t})\leq T_{e,t}\leq T_{e-\max}(\omega_{e,t}) \end{array}\right.$$

Here, *t* is the time variable, $\omega_{m}$ is the motor speed, $T_{m}$ is the motor torque, $\omega_{e}$ is the engine speed, and $T_{e}$ is the engine torque.

The determination of the *SOC* threshold is important for the change of mode, and in this study, the genetic algorithm (GA) [19] is used to optimize the *SOC* threshold. The genetic algorithm optimization process is parameter coding, using a binary coding range of 00000–11111 to code the parameters: initial population setting, selecting a population size of 100 based on empirical parameters, and the maximum number of iterations is 100; selection, crossover, and variation, using the roulette wheel method of selection strategy with a crossover probability of 0.85 and a variation probability of 0.01; fitness function setting, the optimization goal of the genetic algorithm is the optimal energy economy of the whole vehicle, setting the fitness function as:

(12) $$L=\min E_{d}(t)$$

The optimization results in the continuous evolution of the population and the value of the fitness function of the optimal individual decreases continuously and finally converges at approximately 27.5, when the most corresponding individuals $SOC_{down}$ = 0.25 and $SOC_{up}$ = 0.38.

The specific process of the energy management strategy according to the key parameters of the threshold value is as follows:

(1) The battery *SOC* value is greater than $SOC_{up}$ (power consumption Charge Depleting (CD) mode).

① When the demand speed of the vehicle is less than the upper speed limit of the engine operation, or the demand torque of the vehicle is greater than zero and less than the maximum torque range provided by the electric motor, the pure electric drive mode is adopted.

② When the demand speed of the vehicle is greater than the upper speed limit of the engine operation and the demand torque of the vehicle is greater than the maximum torque provided by the electric motor, the hybrid drive mode is adopted, and an instantaneous search is performed according to the energy economy function of the engine and the electric motor to determine the torque distribution of the power source at this time.

(2) Battery SOC value is greater than $SOC_{down}$ and less than $SOC_{up}$ (power maintenance Charge Sustaining (CS) mode).

① When the demand speed of the vehicle is less than the upper limit of the engine working speed or the demand torque of the vehicle is greater than zero and less than the minimum torque provided by the engine high-efficiency zone, the pure electric drive mode is used.

② When the demand speed of the vehicle is greater than the upper limit of the engine working speed and the demand torque of the vehicle is greater than zero and less than the maximum torque that can be provided by the engine high-efficiency zone, the pure engine drive mode is adopted.

③ When the demand speed of the vehicle is greater than the upper limit of the engine working speed and the demand torque of the vehicle is greater than the maximum torque that the engine can provide, the hybrid drive mode is adopted, and an instantaneous search for optimization is performed according to the energy economy function of the engine and the motor to determine the torque distribution of the power source at this time.

(3) The battery SOC value is less than $SOC_{down}$ (traveling charging mode).

In the engine active charging mode, the engine works in the optimal economic curve, with a larger torque to battery active charging, such that the battery quickly returns to the above $SOC_{down}$ value.

The mode switching conditions and torque distribution of the energy management control strategy are listed in Table 3.

### 4.3. The Proposed Real-Time Energy Management

**Figure 14:**
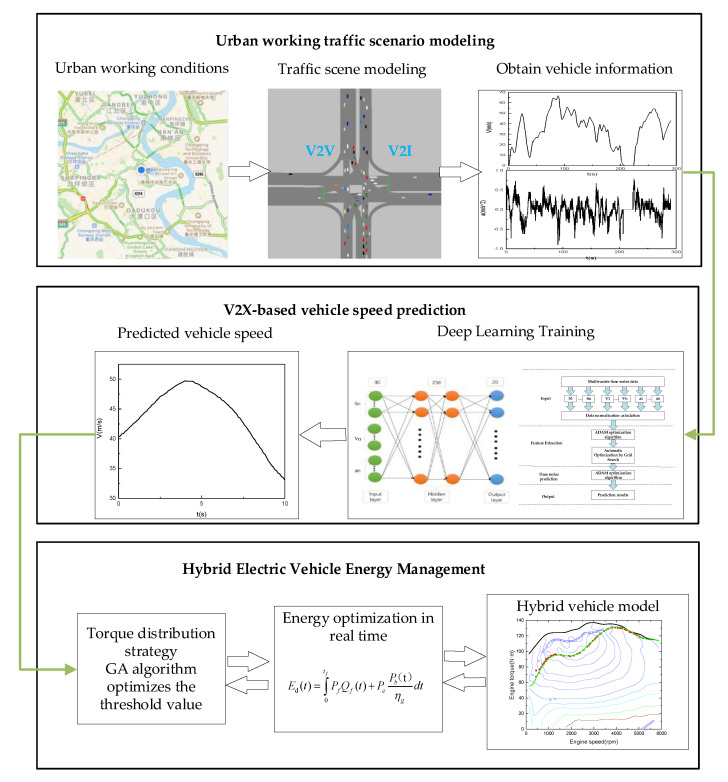
Overall structure of the proposed energy management.

In summary, a real-time energy management, as illustrated in Figure 14, is proposed based on V2X vehicle speed prediction.

As shown in Figure 14, the proposed energy management includes three steps, as follows:

(1) The first step is to establish an urban road network using VISSIM to simulate vehicle V2X communication under urban conditions to obtain real-time vehicle speed and traffic information. The traffic scenario of traffic light intersection is established, and the rules of traffic signal position time, intersection, traffic flow, and vehicle expectation plus or minus speed are set.

(2) The second step is to establish a vehicle speed prediction model using a deep learning neural network to obtain information through V2V and V2I to predict the speed of the target vehicle in the next 10 s.

(3) The third step is to optimize the logic threshold value based on the GA algorithm combined with a real-time optimization algorithm to predict the torque distribution of the whole vehicle in the time domain to achieve optimal fuel economy and reduce carbon emissions.

## 5. Analysis of Simulation Results

**Figure 15:**
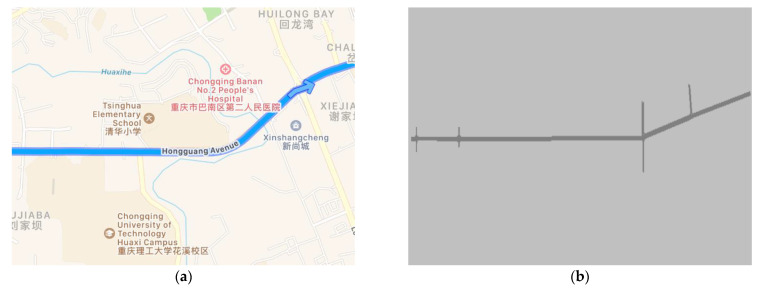
Test road: (**a**) Test road under high precision map; (**b**) Test road under VISSIM scene modeling.

In this study, the road section of Hongguang Avenue from the northeast gate of Chongqing University of Technology to the fork in the direction of the two lanes, as shown in Figure 15a, was selected as the research object to model the traffic scenario and verify the energy management strategy of the hybrid power system based on the V2X speed prediction proposed in the text. In this study, a road of approximately 2.5 km was selected as the test road; the road passed through a total of four traffic light intersections, and the maximum design speed of the road was 60 km/h. The traffic scenario modeling of the road was conducted in Vissim, as shown in Figure 15b.

In the testing process, this study stipulates that all vehicles in the traffic scenario are equipped with V2X communication functions, ignoring the delay, error rate, road slope, and collision of vehicle communication. Real-vehicle data collection is conducted as input conditions, and the whole vehicle model is built based on the MATLAB/Simulink platform to verify the impact of the energy management strategy based on the logic threshold and transient optimization algorithm proposed in this study on the energy economy of the whole vehicle.

### Optimization Results and Analysis

**Figure 16:**
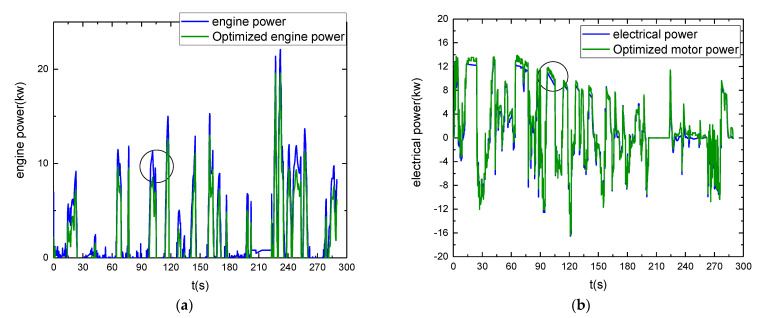
Power variation comparison: (**a**) Comparison of engine output power; (**b**) Motor output power comparison.

**Figure 17:**
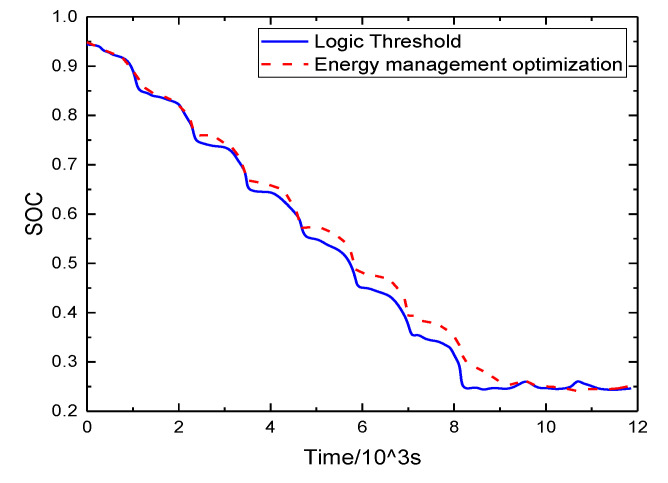
SOC change curve.

According to the above strategy, Figure 16 shows the comparison of engine output power and motor output power, and that the torque distribution under different control strategies is different; thus, the power distribution is different. As in the 90 to 110 s stage, the vehicle speed is high and stable, reducing the engine output power and effectively reducing fuel consumption. From Figure 17, it can be seen that the battery SOC decreases more slowly and fluctuates less after adopting the energy management strategy.

**Figure 18:**
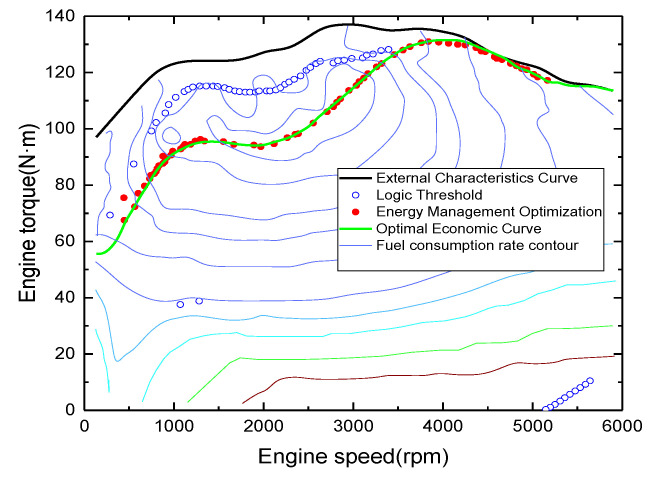
Engine operating point.

**Figure 19:**
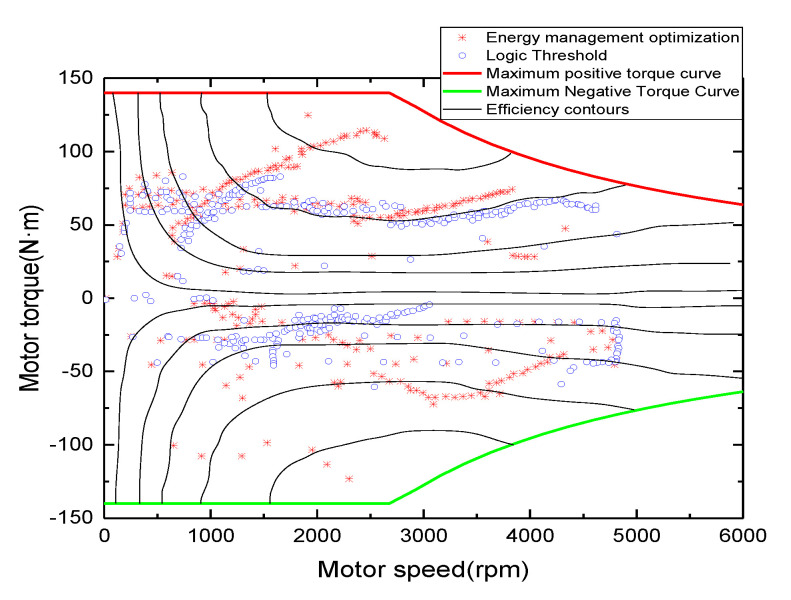
Motor operating point.

The engine and motor operating points were compared and analyzed. From Figure 18 and Figure 19, it can be seen that the operating points of the engine are mostly concentrated near the optimal economy curve after optimization using the energy management strategy, and the fuel consumption is reduced; similarly, the motor is also partially in the relatively high-efficiency operating area. Under the energy management strategy proposed in this study, the total fuel consumption is 2.27 L, the total electricity consumption is 5.42 kwh, and the energy economy is CNY 17.70. Compared with the energy management strategy based on logical thresholds, the proposed energy management strategy reduces fuel consumption by 19.5%, increases electricity consumption by 43.38%, improves energy economy by 13.02%, and reduces CO_2_ emissions by 16.04%. The fuel economy was significantly improved, which again shows that the established energy management control strategy is effective. A comparison of the performance of the two control strategies is shown in Table 4.

## 6. Conclusions

In this study, a plug-in single-axle parallel hybrid vehicle model is built, and the proposed energy management control strategy is validated based on the model, which effectively reduces fuel consumption and improves the energy economy.

A speed prediction model based on a deep learning network was designed to improve the accuracy of vehicle speed prediction. Combined with VISSIM to build a scenario, the speed prediction is performed by simulating the speed and traffic information of the vehicle in front of the target vehicle through V2V and V2I communication technologies under the conditions of intelligent network connection to ensure the real-time accuracy of the energy management strategy.

Subsequently, an energy management control strategy based on a combination of logic threshold and instantaneous optimization is designed to achieve optimal power allocation for hybrid vehicles based on power demand, and the effectiveness of the strategy is verified through actual urban cycling conditions, which significantly improves the energy economy and reduces carbon emissions.

Future work will focus on using deep learning networks to predict the vehicle speed for the entire real smart grid vehicle driving cycle and validate it experimentally. The predicted vehicle speed will be further combined with a globally optimized energy management control strategy to achieve an optimal energy consumption economy for the entire driving cycle.

## Figures and Tables

**Table 1:** Main parameters of hybrid vehicles.

Part	Parameter	Value
Vehicle	Vehicle mass m/kg	1350
	Windward area A/m^2^	2.28
	Air resistance coefficient C_d_	0.32
	Wheel radius r/m	0.295
	Tire rolling resistance coefficient f_r_	0.0135
Engine	Maximum power P_emax_/kw	68
	Maximum torque T_emax_/(N·M)	137
	Rotational speed $\omega_{e}$/(r·min^−1^)	800–6000
Motor	Maximum power P_mmax_/kw	60
	Maximum torque T_mmax_/(N·M)	140
	Rotational speed $\omega_{m}$/(r·min^−1^)	0–6000
Battery	Capacity Q/(A·h)	40
	Nominal voltage U/V	336
CVT	Speed ratio i_cvt_	0.422–2.432
	Main reduction ratio	5.24

**Table 2:** Root mean square error of the deep learning network.

Number of Hidden Layers	Number of Nodes per Layer	Learning Rate	Number of Iterations	RMSE
2	256	0.1	100	0.056996938
2	64	0.001	1000	0.095363609
2	64	0.1	1000	0.095460914
1	512	0.0001	1000	0.097611815
1	256	0.001	1000	0.101234831
2	512	0.1	100	0.103126287
3	512	0.0001	100	0.111647338
1	8	0.0001	10	1.444064856
1	8	0.01	10	1.470003366
1	8	0.1	10	1.497559309

**Table 3:** Energy management control strategy.

Mode	Conditions	Torque Distribution
CD Electric drive	$SOC>SOC_{up}$ $N\leq N_{up-lit}\;or\;0\leq T_{req}\leq T_{ebest}$	$\left\{ \begin{array}{l} T_{m}=T_{req}\\ T_{e}=0 \end{array}\right.$
CD Hybrid drive	$SOC>SOC_{up}$ $N>N_{up-lit};\;T_{req}\geq T_{ebest}$	Instant Advantage Search
CS Electric drive	$SOC_{down}\leq SOC\leq SOC_{up}$ $N\leq N_{up-lit}\;or\;0\leq T_{req}\leq T_{ebest}$	$\left\{ \begin{array}{l} T_{m}=T_{req}\\ T_{e}=0 \end{array}\right.$
CS Engine drive	$SOC_{down}\leq SOC\leq SOC_{up}$ $N>N_{up-lit}\;or\;0\leq T_{req}\leq T_{ebest}$	$\left\{ \begin{array}{l} T_{e}=T_{req}\\ T_{m}=0 \end{array}\right.$
CS Hybrid drive	$SOC_{down}\leq SOC\leq SOC_{up}$ $N>N_{up-lit};\;T_{req}\geq T_{ebest}$	Instant Advantage Search
Charging	$SOC<SOC_{down}$	$\left\{ \begin{array}{l} T_{e}=T_{ebest}\\ T_{m}=T_{e}-T_{req} \end{array}\right.$
Brake Recovery	$T_{req}<0$	$\left\{ \begin{array}{l} T_{m}=T_{req}\\ T_{e}=0 \end{array}\right.$

**Table 4:** Comparison of economic performance on the test road.

Performance Parameters	Fuel Consumption L/100 km	Electricity Consumption kwh/100 km	Energy Economy (CNY)	Economic Improvement (%)	CO_2_ Emissions (g/km)	CO_2_ Emissions Reduction (%)
Based on logical thresholds	2.82	3.78	20.35	-	68.64	-
Based on logic threshold with transient optimization	2.27	5.42	17.7	13.02	57.63	16.04

## Data Availability

Not applicable.
